# Supplementary material for: Fractal Kinetic Implementation in Population Pharmacokinetic Modeling
Source: Pharmaceutics. 2023 Jan 16;15(1):304. doi: 10.3390/pharmaceutics15010304 (PMC9867137; doi:10.3390/pharmaceutics15010304)
Supplement: Supplementary file 1 [file pharmaceutics-15-00304-s001.zip › pharmaceutics-2113163-supplementary.pdf]

pharmaceutics

MDPI

Supplementary Materials: Fractal Kinetic Implementation in Population Pharmacokinetic Modeling

Woojin Jung, Hyo-jeong Ryu, Jung-woo Chae and Hwi-yeol Yun

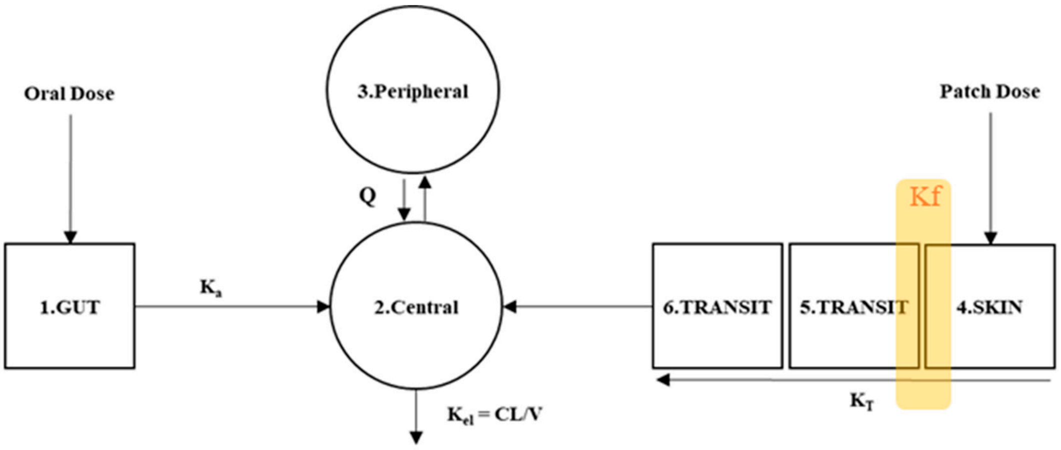

Figure S1. Compartmental scheme of case 1 and fractional rate modification site (highlighted).

Table S1. Estimated results of case 1.

	**Base**		**Fractal**	
**OFV**	**1443.7**		**1410.08**	
**Parameter**	**Estimates (RSE%)**	**IIV in CV% (RSE%) [Shr%]**	**Estimates (RSE%)**	**IIV in CV% (RSE%) [Shr%]**
Ka (1/h)	0.0497 (24.1)	9.86 (55.1) [50.63]	0.0835 (54.4)	15.7 (45.7) [44.85]
CL (L/h)	10 (8.49)	37.3 (24.7) [0.00]	9.68 (8.89)	34.7 (23.7) [0.00]
Vc (L)	26.2 (34)	46.8 (66.6) [42.241]	51.1 (62.3)	41.8 (64.6) [42.73]
Vp (L)	562 (11.4)		564 (9.27)	
Q (L/h)	15.6 (32.4)		28.4 (52.3)	
Kt (1/h)	0.027 (8.91)	14.2 (73.3) [31.38]	0.0439 (21.3)	17.7 (75.9) [30.57]
Add-error	2.89 (12.7)		2.53 (16.5)	
Prop-error	0.0795 (28.7)		0.0909 (25.1)	
h			0.32 (24.3)	

Ka: absorption rate (in fractal model, instantaneous rate is decided with Ka/t^{-h}, unit is 1/time), CL: clearance, Vc: central volume of distribution, Vp: peripheral volume of distribution, Q: inter-compartmental clearance, Kt: transit rate, Add-error: additive error, Prop-error: proportional error, h: heterogeneity exponent. IIV: inter-individual variability, CV: coefficient of variation, RSE: relative standard error, Shr: shrinkage

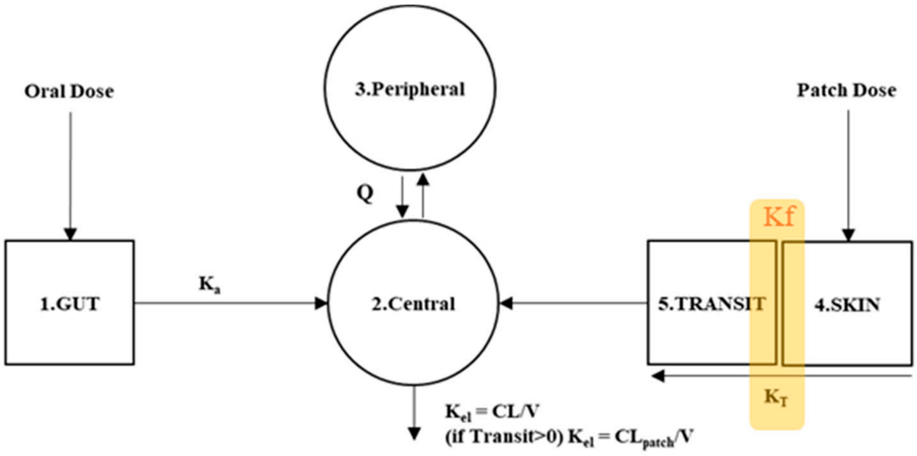

Figure S2. Compartmental scheme of case 2 and fractional rate modification site (highlighted).

Table S2. Estimated results of case 2.

	**Base**		**Fractal**	
**OFV**	**13977.1**		**13592**	
**Parameter**	**Estimates (RSE%)**	**IIV in CV% (RSE%) [Shr%]**	**Estimates (RSE%)**	**IIV in CV% (RSE%) [Shr%]**
Ka (1/h)	0.729 (10.3)	59.5 (42) [29.94]	0.915 (11.9)	51.7 (39) [34.24]
CL (L/h)	9.38 (3.73)	20.8 (17.2) [0.83]	8.97 (3.44)	21.1 (17.1) [0.49]
Vc (L)	196 (10.4)	57.3 (47.8) [12.37]	244 (5.6)	42.1 (35) [12.17]
Vp (L)	526 (5.47)		413 (3.69)	
Q (L/h)	49.8 (7.25)	16.4 (51.3) [57.96]	56.7 (2.51)	7.48 (277) [83.55]
Kt (1/h)	0.0245 (4.76)	48 (26.9) [8.40]	0.0186 (5.15)	33.4 (32) [11.67]
Kf (1/h)	0.0276 (9.21)		0.398 (20.9)	
Add-error	0.293 (8.04)		0.678 (31.9)	
Prop-error	0.186 (7.44)		0.164 (7.7)	
h			0.894 (7.01)	16.5 (27.1) [13]

Ka: absorption rate (in fractal model, instantaneous rate is decided with Ka/t^{-h}, unit is 1/time), CL: clearance, Vc: central volume of distribution, Vp: peripheral volume of distribution, Q: inter-compartmental clearance, Kt: transit rate, Kf: rate from formulation to skin, Add-error: additive error, Prop-error: proportional error, h: heterogeneity exponent. IIV: inter-individual variability, CV: coefficient of variation, RSE: relative standard error, Shr: shrinkage

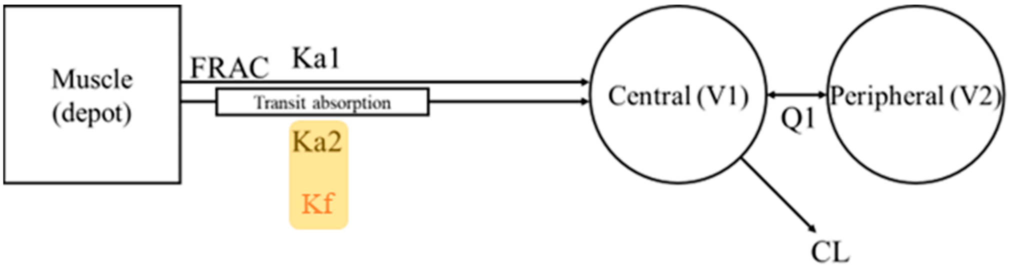

Figure S3. Compartmental scheme of case 3 and fractional rate modification site (highlighted).

Table S3. Estimated results of case 3.

	Base		Fractal	
**OFV**	**2155.43**		**2153.54**	
**Parameter**	**Estimates (RSE%)**	**IIV in CV% (RSE%) [Shr%]**	**Estimates (RSE%)**	**IIV in CV% (RSE%) [Shr%]**
V1 (L)	0.233 (13.6)	39.1 (59.2) [34.21]	0.356	26.6 [45.59]
V2 (L)	1.99 (12.9)		2.62	
CL (L/h)	0.145 (12.3)		0.175	
Q (L/h)	0.408 (21)	43.2 (107) [33.41]	0.66	47.2 [32.69]
Ka1 (1/h)	0.0843 (14)	20.5 (88.3) [22.33]	0.104	19.3 [23.54]
Ka2 (1/h)	0.00171 (12.3)		0.00179	
MTT (h)	135 (1.47)		136	
N (unitless)	86.8 (12.6)	6.91 (71) [27.00]	83.8	8.08 [28.27]
FRAC (unitless)	0.138 (10.8)	44.3 (30.5) [1.58]	0.172	42.1 [0.00]
Add-error			0.133	
Prop-error	0.321 (8.36)		0.323	
h			0.0268	124 [59.25]

V1: central volume of distribution, V2: peripheral volume of distribution, CL: clearance, Q: inter-compartmental clearance, Ka1: fast absorption rate (in fractal model, instantaneous rate is decided with Ka/t^{-h}, unit is 1/time), Ka2: slow absorption rate, MTT: mean transit time, N: number of transit compartment, FRAC: fraction to the fast absorption compartment, Add-error: additive error, Prop-error: proportional error, h: heterogeneity exponent. IIV: inter-individual variability, CV: coefficient of variation, RSE: relative standard error, Shr: shrinkage

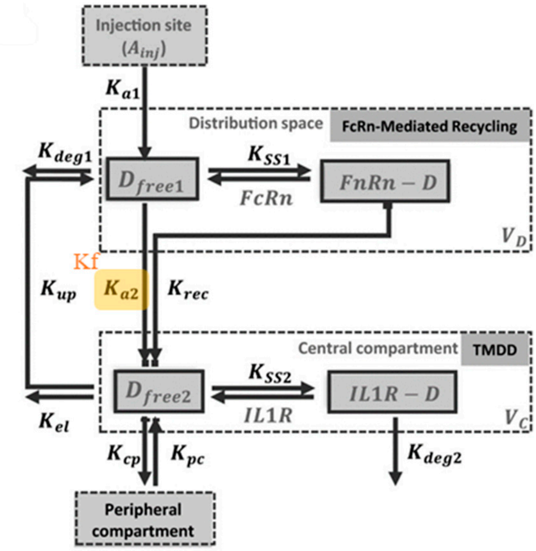

Figure S4. Compartmental scheme of case 4 and fractional rate modification site (highlighted).

Table S4. Estimated results of case 4.

	Base		Fractal	
**OFV**	**1556.43**		**1539.64**	
**Parameter**	**Estimates (RSE%)**	**IIV in CV% (RSE%) [Shr%]**	**Estimates (RSE%)**	**IIV in CV% (RSE%) [Shr%]**
Kinj (1/h)	1.22 (29.4)	127 (56.9) [13.59]	0.723 (33.1)	103 (46) [11.01]
Kss1 (nmol)	246 (107)		426 (129)	
FcRn (nmol)	746 (98.1)		685 (72.7)	
Ka1 (1/h)	0.0168 (39.4)	83.4 (77.3) [12.37]	0.0246 (78.4)	94.9 (87.2) [15.06]
Ka2 (1/h)	0.0341 (53.3)	33.9 (123) [40.29]	0.0472 (49.6)	36.5 (76.4) [33.02]
Kdeg (1/h)	0.0262 (40.9)	26.7 (155) [38.67]	0.0229 (56)	20.9 (190) [45.58]
CL (L/h)	0.21 (36.1)	23.5 (128) [21.00]	0.228 (39.6)	23.5 (115) [16.03]
Vc (L)	11.2 (25.7)		11.3 (27.7)	
Q (L/h)	0.029 (34.1)		0.0304 (38.1)	
Vp (L)	5.06 (fixed)		5.06 (fixed)	
Kint (1/h)	0.206 (fixed)		0.206 (fixed)	
Rtot (nmol/L)	2.16 (76.5)		1.59 (87.5)	
Kss2 (nmol/L)	14.1 (67.6)		11.6 (63.9)	
Kup (1/h)	0.00952 (fixed)	114 (66.2) [24.55]	0.00952 (fixed)	166 (104) [30.22]
Alag (h)	0.314 (28.1)	59.6 (140) [36.90]	0.286 (32.3)	62.4 (139) [32.61]
Add-error	0.184 (35.6)		0.198 (33.7)	
Prop-error	0.115 (4.22)		0.108 (4.81)	
h			0.277 (60.8)	

Kinj: injection rate, Kss1: equilibrium dissociation constant of drug and receoptor binding, FcRn: FcRn receptor conentration, Ka1: absorption rate from absorption site (in fractal model, instantaneous rate is decided with Ka/t^{-h}, unit is 1/time), Ka2: recycled rate from absorption site, Kdeg: degradation rate, CL: clearance, Vc: central volume of distribution, Q: inter-compartmental clearance, Vp: peripheral volume of distribution, Kint: internalization rate consant, Rtot: total receptor concentration, Kss2: Dissociation constant of drug and target binding, Kup: Uptake rate constant of drug from central back to absorption site, Alag: lag-time, Add-error: additive error, Prop-error: proportional error, h: heterogeneity exponent. IIV: inter-individual variability, CV: coefficient of variation, RSE: relative standard error, Shr: shrinkage

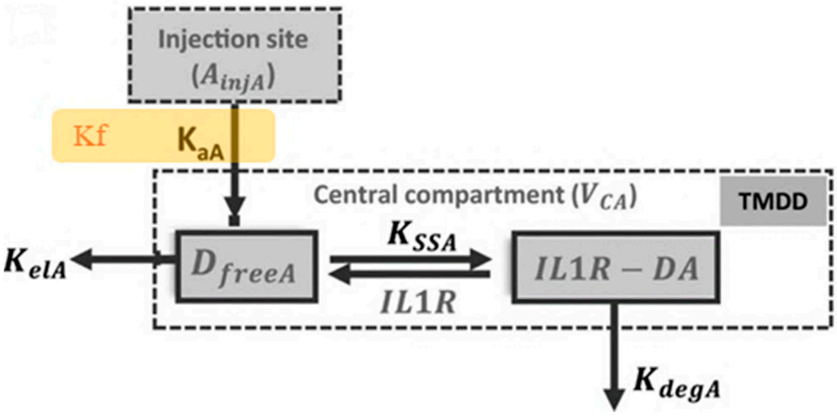

Figure S5. Compartmental scheme of case 5 and fractional rate modification site (highlighted).

Table S5. Estimated results of case 5.

	Base		Fractal	
**OFV**	**358.476**		**350.131**	
**Parameter**	**Estimates (RSE%)**	**IIV in CV% (RSE%) [Shr%]**	**Estimates (RSE%)**	**IIV in CV% (RSE%) [Shr%]**
CL (L/h)	9.61 (5.22)	10.3 (55.5) [0.00]	9.03	11.1 [0.00]
Vc (L)	19.4 (20)	40.7 (61.9) [0.98]	53.7	20.1 [0.00]
Ka (1/h)	0.167 (12.7)	25.4 (55.9) [0.00]	0.47	27 [24.59]
Kint (1/h)	0.206 (fixed)		0.206 (fixed)	
Rtot (nmol/L)	1.68 (98.9)		1.67	
Kss (nmol/L)	0.521 (68.6)		1.39	
Add-error	0.0606 (64.7)		0.0294	
Prop-error	0.114 (8.8)		0.113	
h			0.139	157 [9.87]

CL: clearance, Vc: central volume of distribution, Ka: absorption rate, Kint: internalization rate consant (in fractal model, instantaneous rate is decided with Ka/t^{-h}, unit is 1/time), Rtot: total receptor concentration, Kss: quasi-steady sate constant for interactions of IL1R and anakinra, Add-error: additive error, Prop-error: proportional error, h: heterogeneity exponent. IIV: inter-individual variability, CV: coefficient of variation, RSE: relative standard error, Shr: shrinkage

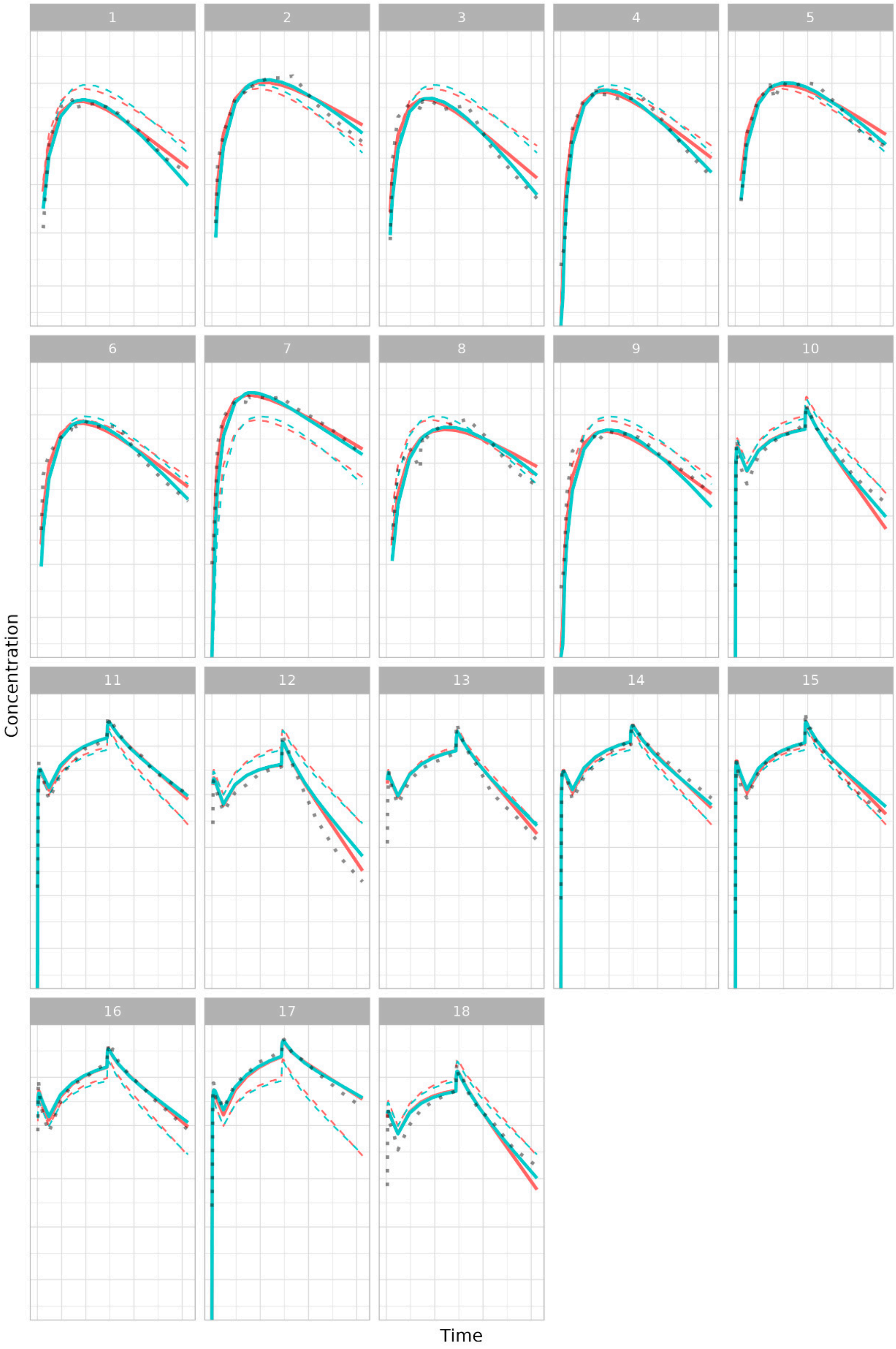

Figure S6. Individual plot of case 1. Black dotted line: observation, dashed line: population prediction, solid line: individual prediction, blue lines: base model, red lines: fractal model.

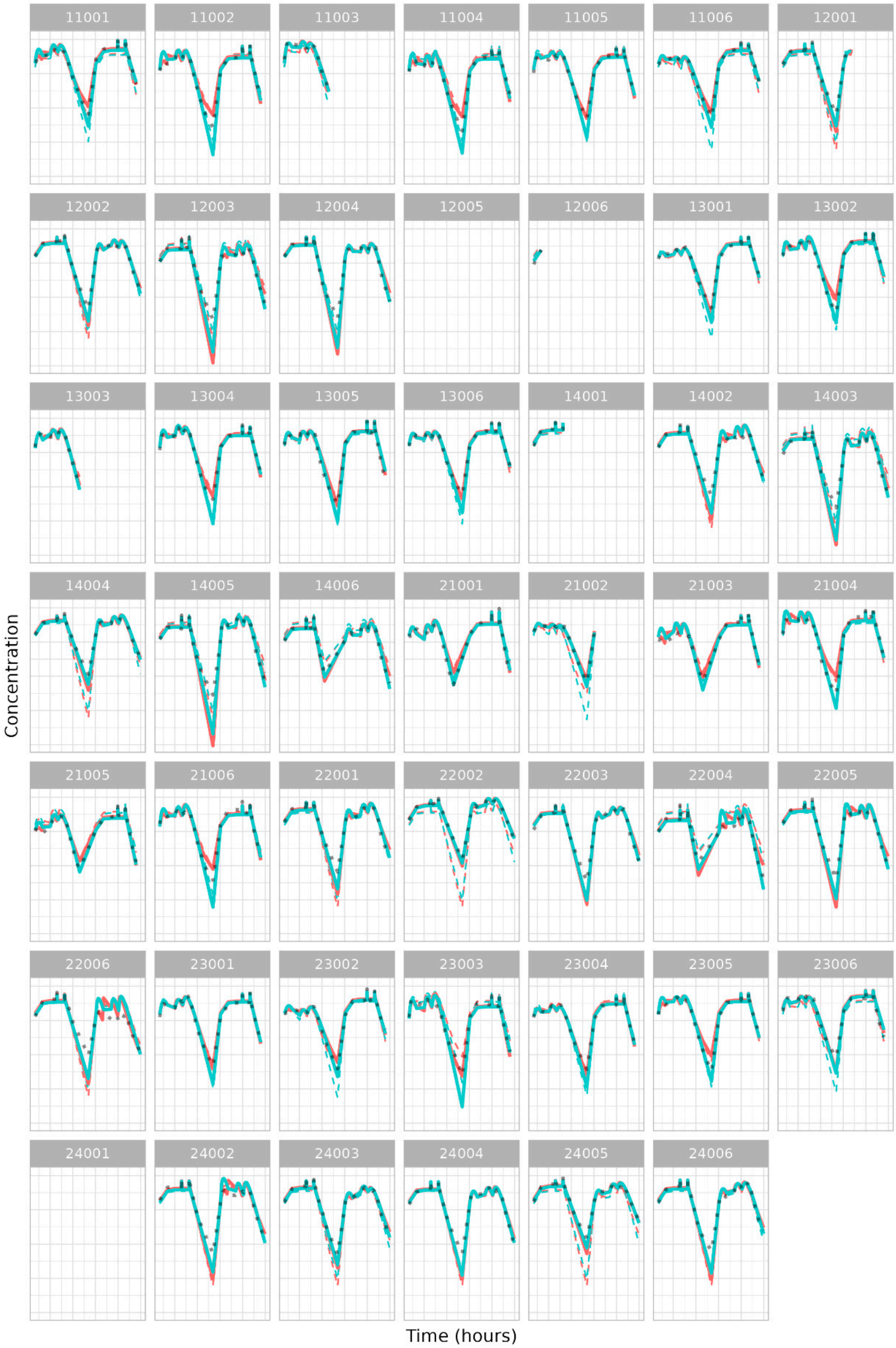

Figure S7. Individual plot of case 2. Black dotted line: observation, dashed line: population prediction, solid line: individual prediction, blue lines: base model, red lines: fractal model.

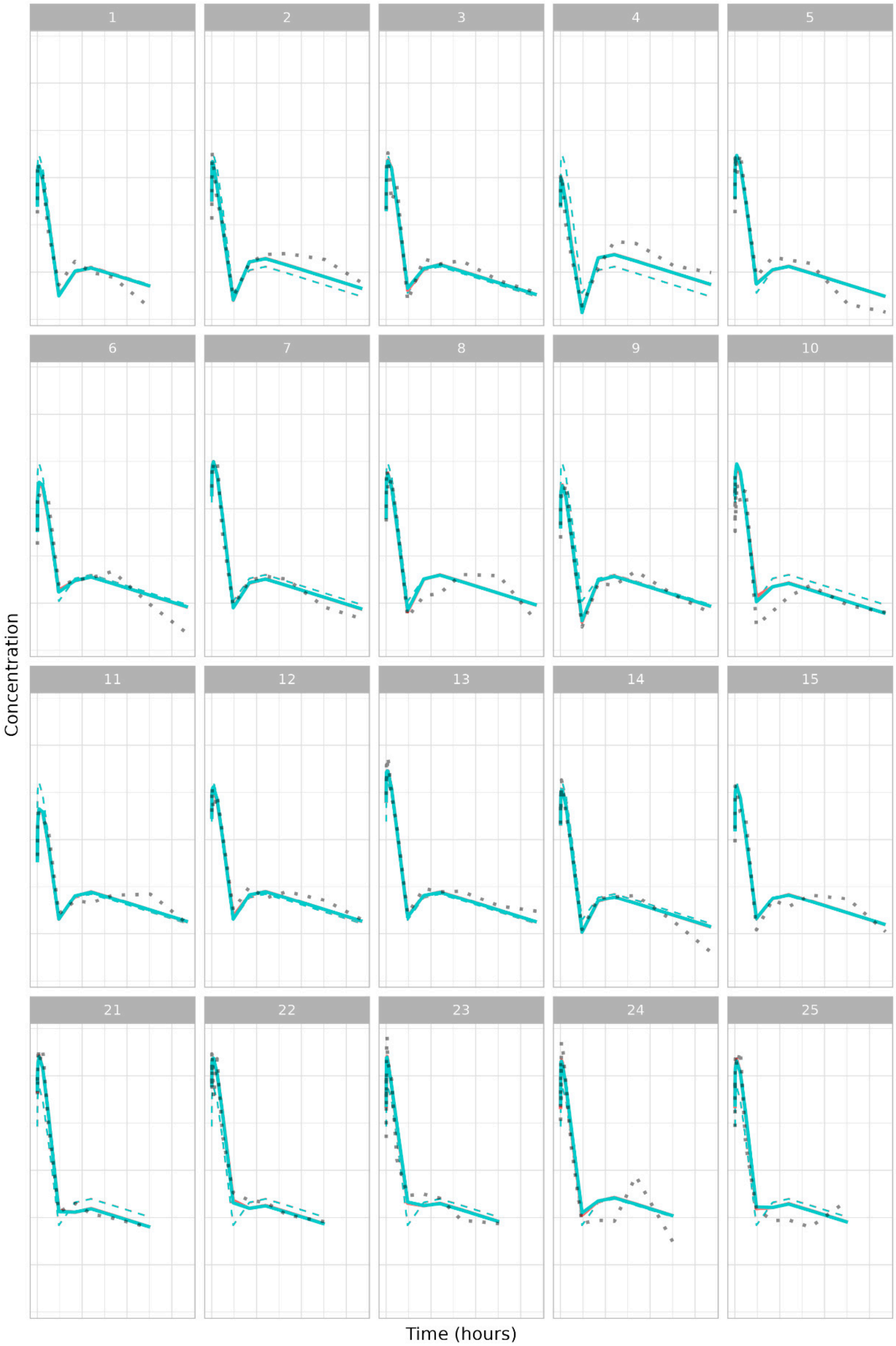

Figure S8. Individual plot of case 3. Black dotted line: observation, dashed line: population prediction, solid line: individual prediction, blue lines: base model, red lines: fractal model.

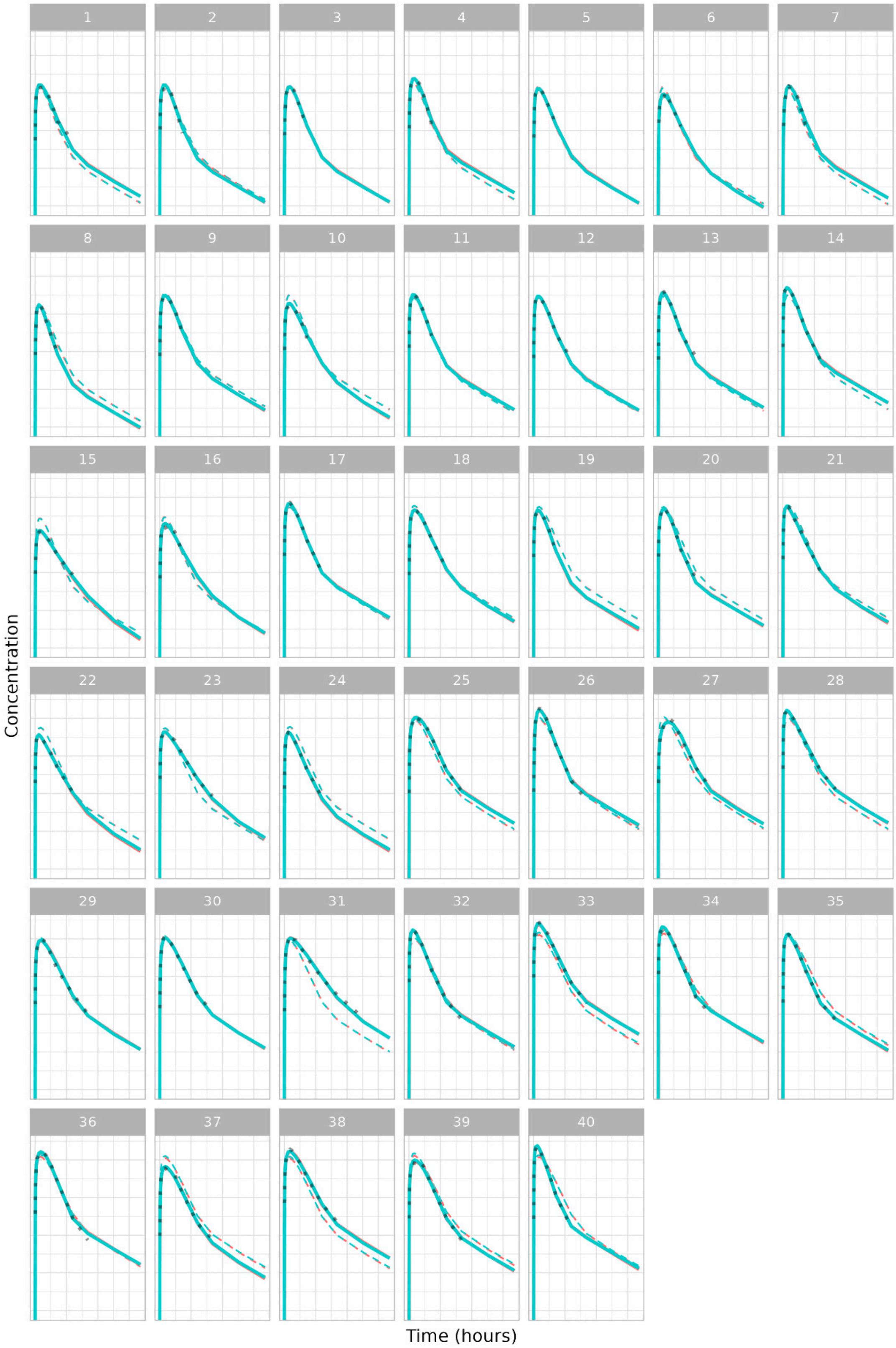

Figure S9. Individual plot of case 4. Black dotted line: observation, dashed line: population prediction, solid line: individual prediction, blue lines: base model, red lines: fractal model.

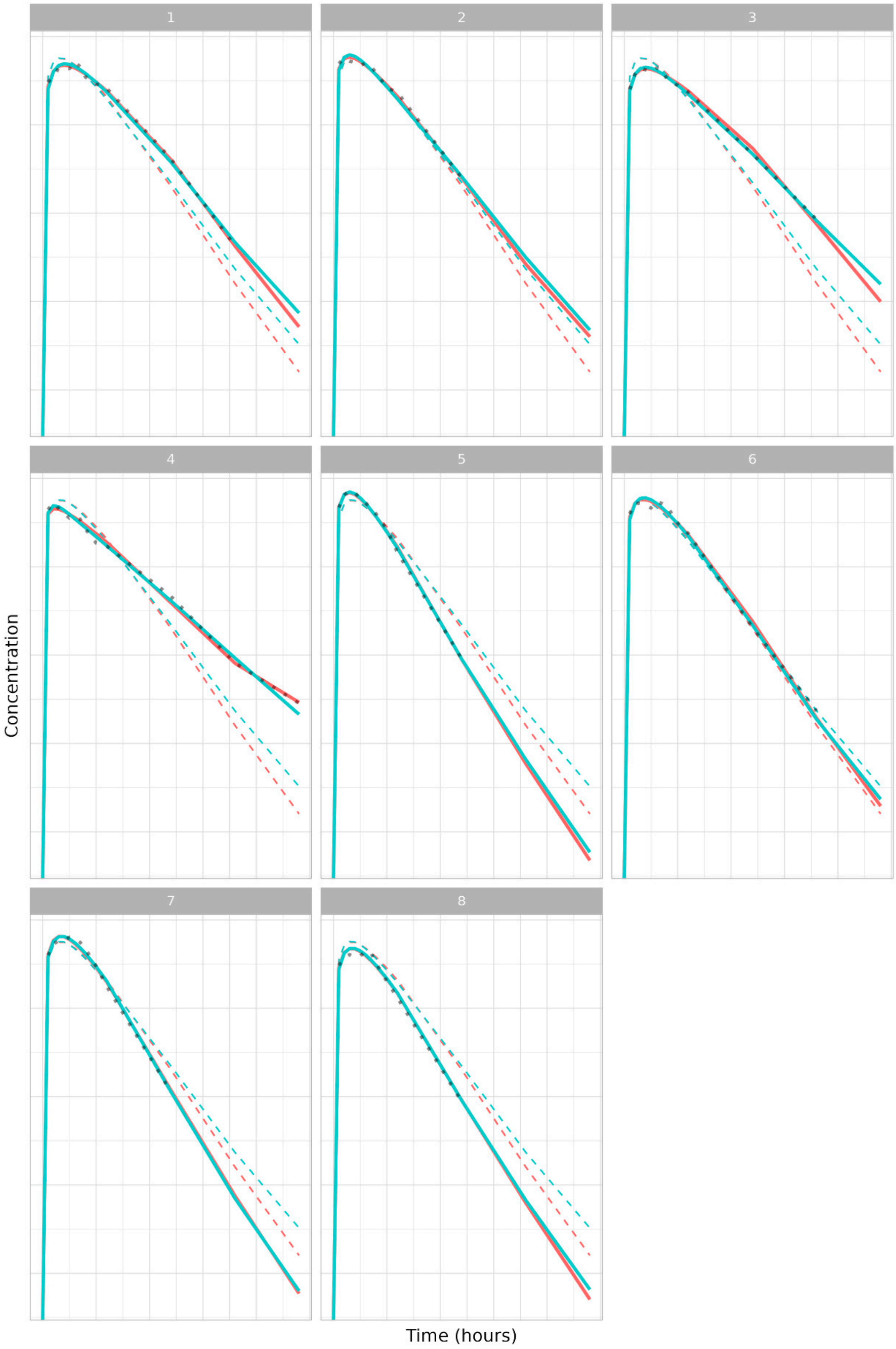

Figure S10. Individual plot of case 5. Black dotted line: observation, dashed line: population prediction, solid line: individual prediction, blue lines: base model, red lines: fractal model.

Code S1. R Code for simulation.

```
library(rxode2)

library(ggplot2)

library(dplyr)

library(xpose)


# simulation ==============================================================

# event table for simulation --------------------------------------------------

ev_s <- eventTable()

ev_s$add.dosing(dose=100, dosing.to = 1, dosing.interval = 50, nbr.doses = 1)

ev_s$add.sampling(seq(0,400, by=1))


ev_m <- eventTable()

ev_m$add.dosing(dose=100, dosing.to = 1, dosing.interval = 50, nbr.doses = 15)

ev_m$add.sampling(seq(0,400, by=1))


# 1 compartment model ---------------------------------------------------------

mod_1comp_abs <- RxODE({

  CP = centr/V1

  KE = CL/V1


  d/dt(depot) = -KA*(time**(-h))*depot

  d/dt(centr) = KA*(time**(-h))*depot - KE*centr


})


mod_1comp_ke <- RxODE({

  CP = centr/V1
```

```
  KE = CL/V1


  d/dt(depot) = -KA*depot

  d/dt(centr) = KA*depot - KE*(time**(-h))*centr


})


# 2 compartment model ---------------------------------------------------------

mod_2comp_abs <- RxODE({

  CP = centr/V1

  KE = CL/V1


  K12 = Q/V1

  K21 = Q/V2


  d/dt(depot) = -KA*(time**(-h))*depot

  d/dt(centr) = KA*(time**(-h))*depot - K12*centr + K21*peri - KE*centr

  d/dt(peri) = K12*centr - K21*peri

})


mod_2comp_ke <- RxODE({

  CP = centr/V1

  KE = CL/V1


  K12 = Q/V1

  K21 = Q/V2


  d/dt(depot) = -KA*depot
```

```
  d/dt(centr) = KA*depot - K12*centr + K21*peri - KE*(time**(-h))*centr
  d/dt(peri) = K12*centr - K21*peri
})

mod_2comp_k12 <- RxODE({
  CP = centr/V1
  KE = CL/V1

  K12 = Q/V1
  K21 = Q/V2

  d/dt(depot) = -KA*depot
  d/dt(centr) = KA*depot - K12*(time**(-h))*centr + K21*peri - KE*centr
  d/dt(peri) = K12*(time**(-h))*centr - K21*peri
})

mod_2comp_k21 <- RxODE({
  CP = centr/V1
  KE = CL/V1

  K12 = Q/V1
  K21 = Q/V2

  d/dt(depot) = -KA*depot
  d/dt(centr) = KA*depot - K12*centr + K21*(time**(-h))*peri - KE*centr
  d/dt(peri) = K12*centr - K21*(time**(-h))*peri
})
```

```
fract_plot <- function(model, ev){

  df <- NULL

  n = 20

  for (i in 0:n) {

    theta <- c(model_theta, h=0.05*i)

    temp <- as.data.frame(model$solve(theta,ev))

    temp$iter <- 0.05*i

    df <- rbind(df,temp)

  }

  df

}


model_theta <- c(CL=0.3, Q=3, V1=10, V2=100, KA=0.033)

# Ka < CL, Kcp > Kpc

p1.1 <- fract_plot(mod_1comp_abs, ev_s) %>% mutate(Comp = "1 Comp", param = "Ka",
Cond1 = "Ka < CL", Cond2 = "Kcp > Kpc") %>% select(time,CP,iter,Comp, param,
Cond1, Cond2)

p2.1 <- fract_plot(mod_1comp_ke, ev_s) %>% mutate(Comp = "1 Comp", param = "Ke",
Cond1 = "Ka < CL", Cond2 = "Kcp > Kpc") %>% select(time,CP,iter,Comp, param,
Cond1, Cond2)

p3.1 <- fract_plot(mod_2comp_abs, ev_s) %>% mutate(Comp = "2 Comp", param = "Ka",
Cond1 = "Ka < CL", Cond2 = "Kcp > Kpc") %>% select(time,CP,iter,Comp, param,
Cond1, Cond2)

p4.1 <- fract_plot(mod_2comp_ke, ev_s) %>% mutate(Comp = "2 Comp", param = "Ke",
Cond1 = "Ka < CL", Cond2 = "Kcp > Kpc") %>% select(time,CP,iter,Comp, param,
Cond1, Cond2)

p6.1 <- fract_plot(mod_2comp_k12, ev_s) %>% mutate(Comp = "2 Comp", param =
"Kcp", Cond1 = "Ka < CL", Cond2 = "Kcp > Kpc") %>% select(time,CP,iter,Comp,
param, Cond1, Cond2)

p5.1 <- fract_plot(mod_2comp_k21, ev_s) %>% mutate(Comp = "2 Comp", param =
"Kpc", Cond1 = "Ka < CL", Cond2 = "Kcp > Kpc") %>% select(time,CP,iter,Comp,
param, Cond1, Cond2)
```

```
# KA == CL, Kcp > Kpc

model_theta <- c(CL=0.1, Q=3, V1=10, V2=100, KA=0.1)

p1.2 <- fract_plot(mod_1comp_abs, ev_s) %>% mutate(Comp = "1 Comp", param = "Ka",
Cond1 = "Ka = CL", Cond2 = "Kcp > Kpc") %>% select(time,CP,iter,Comp, param,
Cond1, Cond2)

p2.2 <- fract_plot(mod_1comp_ke, ev_s) %>% mutate(Comp = "1 Comp", param = "Ke",
Cond1 = "Ka = CL", Cond2 = "Kcp > Kpc") %>% select(time,CP,iter,Comp, param,
Cond1, Cond2)

p3.2 <- fract_plot(mod_2comp_abs, ev_s) %>% mutate(Comp = "2 Comp", param = "Ka",
Cond1 = "Ka = CL", Cond2 = "Kcp > Kpc") %>% select(time,CP,iter,Comp, param,
Cond1, Cond2)

p4.2 <- fract_plot(mod_2comp_ke, ev_s) %>% mutate(Comp = "2 Comp", param = "Ke",
Cond1 = "Ka = CL", Cond2 = "Kcp > Kpc") %>% select(time,CP,iter,Comp, param,
Cond1, Cond2)

p6.2 <- fract_plot(mod_2comp_k12, ev_s) %>% mutate(Comp = "2 Comp", param =
"Kcp", Cond1 = "Ka = CL", Cond2 = "Kcp > Kpc") %>% select(time,CP,iter,Comp,
param, Cond1, Cond2)

p5.2 <- fract_plot(mod_2comp_k21, ev_s) %>% mutate(Comp = "2 Comp", param =
"Kpc", Cond1 = "Ka = CL", Cond2 = "Kcp > Kpc") %>% select(time,CP,iter,Comp,
param, Cond1, Cond2)


# Ka > CL, Kcp > Kpc

model_theta <- c(CL=0.033, Q=3, V1=10, V2=100, KA=0.3)

p1.3 <- fract_plot(mod_1comp_abs, ev_s) %>% mutate(Comp = "1 Comp", param = "Ka",
Cond1 = "Ka > CL", Cond2 = "Kcp > Kpc") %>% select(time,CP,iter,Comp, param,
Cond1, Cond2)

p2.3 <- fract_plot(mod_1comp_ke, ev_s) %>% mutate(Comp = "1 Comp", param = "Ke",
Cond1 = "Ka > CL", Cond2 = "Kcp > Kpc") %>% select(time,CP,iter,Comp, param,
Cond1, Cond2)

p3.3 <- fract_plot(mod_2comp_abs, ev_s) %>% mutate(Comp = "2 Comp", param = "Ka",
Cond1 = "Ka > CL", Cond2 = "Kcp > Kpc") %>% select(time,CP,iter,Comp, param,
Cond1, Cond2)
```

```
p4.3 <- fract_plot(mod_2comp_ke, ev_s) %>% mutate(Comp = "2 Comp", param = "Ke", Cond1 = "Ka > CL", Cond2 = "Kcp > Kpc") %>% select(time,CP,iter,Comp, param, Cond1, Cond2)

p6.3 <- fract_plot(mod_2comp_k12, ev_s) %>% mutate(Comp = "2 Comp", param = "Kcp", Cond1 = "Ka > CL", Cond2 = "Kcp > Kpc") %>% select(time,CP,iter,Comp, param, Cond1, Cond2)

p5.3 <- fract_plot(mod_2comp_k21, ev_s) %>% mutate(Comp = "2 Comp", param = "Kpc", Cond1 = "Ka > CL", Cond2 = "Kcp > Kpc") %>% select(time,CP,iter,Comp, param, Cond1, Cond2)




model_theta <- c(CL=0.3, Q=3, V1=10, V2=1, KA=0.033)

# Ka < CL, Kcp < Kpc

p3.4 <- fract_plot(mod_2comp_abs, ev_s) %>% mutate(Comp = "2 Comp", param = "Ka", Cond1 = "Ka < CL", Cond2 = "Kcp < Kpc") %>% select(time,CP,iter,Comp, param, Cond1, Cond2)

p4.4 <- fract_plot(mod_2comp_ke, ev_s) %>% mutate(Comp = "2 Comp", param = "Ke", Cond1 = "Ka < CL", Cond2 = "Kcp < Kpc") %>% select(time,CP,iter,Comp, param, Cond1, Cond2)

p6.4 <- fract_plot(mod_2comp_k12, ev_s) %>% mutate(Comp = "2 Comp", param = "Kcp", Cond1 = "Ka < CL", Cond2 = "Kcp < Kpc") %>% select(time,CP,iter,Comp, param, Cond1, Cond2)

p5.4 <- fract_plot(mod_2comp_k21, ev_s) %>% mutate(Comp = "2 Comp", param = "Kpc", Cond1 = "Ka < CL", Cond2 = "Kcp < Kpc") %>% select(time,CP,iter,Comp, param, Cond1, Cond2)


# KA == CL, Kcp < Kpc

model_theta <- c(CL=0.1, Q=3, V1=10, V2=1, KA=0.1)

p3.5 <- fract_plot(mod_2comp_abs, ev_s) %>% mutate(Comp = "2 Comp", param = "Ka", Cond1 = "Ka = CL", Cond2 = "Kcp < Kpc") %>% select(time,CP,iter,Comp, param, Cond1, Cond2)

p4.5 <- fract_plot(mod_2comp_ke, ev_s) %>% mutate(Comp = "2 Comp", param = "Ke", Cond1 = "Ka = CL", Cond2 = "Kcp < Kpc") %>% select(time,CP,iter,Comp, param, Cond1, Cond2)
```

```
p6.5 <- fract_plot(mod_2comp_k12, ev_s) %>% mutate(Comp = "2 Comp", param =
"Kcp", Cond1 = "Ka = CL", Cond2 = "Kcp < Kpc") %>% select(time,CP,iter,Comp,
param, Cond1, Cond2)

p5.5 <- fract_plot(mod_2comp_k21, ev_s) %>% mutate(Comp = "2 Comp", param =
"Kpc", Cond1 = "Ka = CL", Cond2 = "Kcp < Kpc") %>% select(time,CP,iter,Comp,
param, Cond1, Cond2)


# Ka > CL, Kcp < Kpc

model_theta <- c(CL=0.033, Q=3, V1=10, V2=1, KA=0.3)

p3.6 <- fract_plot(mod_2comp_abs, ev_s) %>% mutate(Comp = "2 Comp", param = "Ka",
Cond1 = "Ka > CL", Cond2 = "Kcp < Kpc") %>% select(time,CP,iter,Comp, param,
Cond1, Cond2)

p4.6 <- fract_plot(mod_2comp_ke, ev_s) %>% mutate(Comp = "2 Comp", param = "Ke",
Cond1 = "Ka > CL", Cond2 = "Kcp < Kpc") %>% select(time,CP,iter,Comp, param,
Cond1, Cond2)

p6.6 <- fract_plot(mod_2comp_k12, ev_s) %>% mutate(Comp = "2 Comp", param =
"Kcp", Cond1 = "Ka > CL", Cond2 = "Kcp < Kpc") %>% select(time,CP,iter,Comp,
param, Cond1, Cond2)

p5.6 <- fract_plot(mod_2comp_k21, ev_s) %>% mutate(Comp = "2 Comp", param =
"Kpc", Cond1 = "Ka > CL", Cond2 = "Kcp < Kpc") %>% select(time,CP,iter,Comp,
param, Cond1, Cond2)


p <- rbind(

  p3.1, p4.1, p5.1, p6.1,

  p3.2, p4.2, p5.2, p6.2,

  p3.3, p4.3, p5.3, p6.3,

  p3.4, p4.4, p5.4, p6.4,

  p3.5, p4.5, p5.5, p6.5,

  p3.6, p4.6, p5.6, p6.6

)

p$param <- factor(p$param, levels = c("Ka", "Ke", "Kcp", "Kpc"))

p$Cond2 <- factor(p$Cond2, levels = c("Kcp < Kpc", "Kcp > Kpc"))
```

```
ggplot(p) +
  scale_colour_gradient(low = "#66CCCC", high = "#FF6666") +
  geom_line(aes(x=time, y=CP, group=iter, color=iter), alpha=0.3) +
     geom_line(data=dplyr::filter(p,iter==0),aes(x=time,    y=CP,    group=iter),
color="#66CCCC", size=0.6, linetype="twodash") +
    geom_line(data=dplyr::filter(p,iter==0.25),aes(x=time,   y=CP,   group=iter),
color="#66CCCC", size=0.6, linetype="dotted") +
    geom_line(data=dplyr::filter(p,iter==0.5),aes(x=time,   y=CP,   group=iter),
color="#FFCC00", size=0.6) +
    geom_line(data=dplyr::filter(p,iter==0.75),aes(x=time,   y=CP,   group=iter),
color="#FF6666", size=0.6, linetype="dotted") +
     geom_line(data=dplyr::filter(p,iter==1),aes(x=time,    y=CP,    group=iter),
color="#FF6666", size=0.6, linetype="twodash") +

  facet_grid(param ~ Cond2 + Cond1, scales="free") +
  theme_light() +
  xlab("Time (hour)") +
  ylab("Simulated Concentration (mcg/L)") +
  theme(legend.position = "bottom",
        panel.grid.minor.y = element_blank(),
        panel.grid.minor.x = element_blank()) +
  guides(color = guide_legend(title="Heterogeneity (h)",
                              override.aes = list(alpha=1,
                                                                          color  =
c("#66CCCC","#66CCCC","#FFCC00","#FF6666","#FF6666"),
                                                  linetype = c("twodash","dot-
ted","solid","dotted","twodash")
                              )))
```

```
ggsave(filename="sim_frac_2comp.png", width=20, height=15, units="cm", type =
"cairo")



p <- rbind(
  p1.1, p2.1,
  p1.2, p2.2,
  p1.3, p2.3
)
p$param <- factor(p$param, levels = c("Ka", "Ke", "K12", "K21"))


ggplot(p) +
  scale_colour_gradient(low = "#66CCCC", high = "#FF6666",
                        labels = c(0,0.25,0.5,0.75,1)) +
  geom_line(aes(x=time, y=CP, group=iter, color=iter), alpha=0.3) +
    geom_line(data=dplyr::filter(p,iter==0),aes(x=time,   y=CP,   group=iter),
color="#66CCCC", size=0.6, linetype="twodash") +
   geom_line(data=dplyr::filter(p,iter==0.25),aes(x=time,  y=CP,  group=iter),
color="#66CCCC", size=0.6, linetype="dotted") +
    geom_line(data=dplyr::filter(p,iter==0.5),aes(x=time,   y=CP,  group=iter),
color="#FFCC00", size=0.6) +
   geom_line(data=dplyr::filter(p,iter==0.75),aes(x=time,  y=CP,  group=iter),
color="#FF6666", size=0.6, linetype="dotted") +
    geom_line(data=dplyr::filter(p,iter==1),aes(x=time,   y=CP,   group=iter),
color="#FF6666", size=0.6, linetype="twodash") +
  facet_grid(param ~ Cond1, scales="free") +
  theme_light() +
  xlab("Time (hour)") +
  ylab("Simulated Concentration (mcg/L)") +
  theme(legend.position = "bottom",
```

```
        panel.grid.minor.y = element_blank(),
        panel.grid.minor.x = element_blank()) +
  guides(color = guide_legend(title="Heterogeneity (h)",
                              override.aes = list(alpha=1,
                                                                          color =
c("#66CCCC","#66CCCC","#FFCC00","#FF6666","#FF6666"),
                                                    linetype = c("twodash","dot-
ted","solid","dotted","twodash")
                                                   )))



ggsave(filename="sim_frac_1comp.png", width=20, height=10, units="cm", type =
"cairo")
```

Code S2. NONMEM Code for case 1 (base model).

```
;; 1. Based on: P_O_TRANSIT6

;; 2. Description: case1, base

;; x1. Author: Woojin Jung

;; 3. Label: Oral and patch admin combined into two-compartment model

$PROBLEM    CASE1

$INPUT      ID TIME DV LNDV AMT DOSE ROUTE MDV II ADDL CMT FORM

$DATA       data.csv IGNORE=#

$SUBROUTINE ADVAN6 TOL=9


$MODEL

; Oral

COMP = (GUT)      ;1. Gut

COMP = (CENT)      ;2. Central

COMP = (PERI)      ;3. Peripheral

; Patch

COMP = (SKIN)      ;4. Skin

COMP = (TRAN1)      ;5. Depot, from skin, transit 1

COMP = (TRAN2)      ;6. transit 2


$PK

; Oral comp. params ==========

KA = EXP(LOG(THETA(1)) + ETA(1)) ; Absorption rate

CL = EXP(LOG(THETA(2)) + ETA(2)) ; Central clearance

VC = EXP(LOG(THETA(3)) + ETA(3)) ; Vd central

VP = EXP(LOG(THETA(4)))          ; Vd peripheral

Q = EXP(LOG(THETA(5)))           ; Inter-compartmental clearance
```

```
KE=CL/VC                           ; Elimination rate constant

KCP = Q/VC

KPC = Q/VP


; Patch comp. params =========

KT = EXP(LOG(THETA(6)) + ETA(4)) ; n + 1 / MTT


$DES

; Oral compartment diff. equations ==========

DADT(1) = -KA*A(1)                                 ; Depot (gut)

DADT(2) = KA*A(1) + KPC*A(3) - KCP*A(2) - KE*A(2) + KT*A(6); Central_comp

DADT(3) = KCP*A(2) - KPC*A(3)       ; Peripheral_comp


; Patch compartment diff. equations =========

DADT(4) = - KT*A(4)                 ; Skin (formulation)

DADT(5) = KT*A(4) - KT*A(5)         ; Depot, transit 1

DADT(6) = KT*A(5) - KT*A(6)         ; transit 2


$ERROR

IPRED=0

IF(A(2).GT.0) IPRED = A(2)/(VC/1000) ;IPRED


W       =  SQRT(THETA(7)**2 + (THETA(8)**2)*IPRED**2)

IRES    =  IPRED - DV

IWRES   =  IRES/W

Y = IPRED + W*EPS(1)


$THETA
```

```
 (0,0.0496711) ; 1.KA_Oral
 (0,10.0268) ; 2.CL_Central
 (0,26.2189) ; 3.Vd_Central
 (0,562.037) ; 4.Vd_Peri
 (0,15.6292) ; 5.Q
 (0,0.0270191) ; 6.KT
 (0,2.89074) ; 7.Additive error
 (0,0.0795173) ; 8.Proportional error
$OMEGA
 0.00968106  ;   1.IIV KA
 0.130076  ; 2.IIV CL_Central
 0.197923  ;   3.IIV VC
 0.020045  ;   4.IIV KT
$SIGMA  1  FIX
```

Code S3. NONMEM Code for case 1 (fractal model).

```
;; 1. Based on: P_O_TRANSIT6

;; 2. Description: case1, base

;; x1. Author: Woojin Jung

;; 3. Label: Oral and patch admin combined into two-compartment model

$PROBLEM    CASE1

$INPUT      ID TIME DV LNDV AMT DOSE ROUTE MDV II ADDL CMT FORM

$DATA       data.csv IGNORE=#

$SUBROUTINE ADVAN6 TOL=6


$MODEL

; Oral

COMP = (GUT)      ;1. Gut

COMP = (CENT)      ;2. Central

COMP = (PERI)      ;3. Peripheral

; Patch

COMP = (SKIN)      ;4. Skin

COMP = (TRAN1)      ;5. Depot, from skin, transit 1

COMP = (TRAN2)      ;6. transit 2


$PK

; Oral comp. params ==========

KA = EXP(LOG(THETA(1)) + ETA(1)) ; Absorption rate

CL = EXP(LOG(THETA(2)) + ETA(2)) ; Central clearance

VC = EXP(LOG(THETA(3)) + ETA(3)) ; Vd central

VP = EXP(LOG(THETA(4)))          ; Vd peripheral

Q = EXP(LOG(THETA(5)))           ; Inter-compartmental clearance
```

```
KE=CL/VC                          ; Elimination rate constant
KCP = Q/VC
KPC = Q/VP

; Patch comp. params =========
H = THETA(6); fractal exponent
KT = THETA(7) * EXP(ETA(4)) ; rate, transit
KF = EXP(LOG(KT) - H*LOG(TIME))

$DES
; Oral compartment diff. equations ==========
DADT(1) = -KA*A(1)                                 ; Depot (gut)
DADT(2) = KA*A(1) + KPC*A(3) - KCP*A(2) - KE*A(2) + KT*A(6)   ; Central_comp
DADT(3) = KCP*A(2) - KPC*A(3)       ; Peripheral_comp

; Patch compartment diff. equations =========
DADT(4) = - KF*A(4)                    ; Skin (formulation)
DADT(5) = KF*A(4) - KT*A(5)            ; Depot, transit 1
DADT(6) = KT*A(5) - KT*A(6)            ; Depot, transit 1

$ERROR
IPRED=0
IF(A(2).GT.0) IPRED = A(2)/(VC/1000) ;IPRED

W       =  SQRT(THETA(8)**2 + (THETA(9)**2)*IPRED**2)
IRES    =  IPRED - DV
IWRES   =  IRES/W
Y = IPRED + W*EPS(1)
```

```
$THETA
 (0,0.0834578) ; 1.KA_Oral
 (0,9.68116) ; 2.CL_Central
 (0,51.0518) ; 3.Vd_Central
 (0,564.22) ; 4.Vd_Peri
 (0,28.4) ; 5.Q
 (0,0.320373,1) ; 6.h,fractal coefficient
 (0,0.043948,1) ; 7.KT
 (0,2.53394) ; 8.Additive error
 (0,0.0909088) ; 9.Proportional error
$OMEGA
 0.0241974  ;   1.IIV KA
 0.114149  ; 2.IIV CL_Central
 0.161231  ;   3.IIV VC
 0.0309662  ;   4.IIV KT
$SIGMA  1  FIX
```

Code S4. NONMEM Code for case 2 (base model).

```
;; 1. Based on: transit_comp_v1.1

;; 2. Description: case2, base model

;; x1. Author: Woojin Jung

;; 3. Label: Oral and patch admin combined into two-compartment model

$PROBLEM    CASE2

$INPUT      ID TIME INIT REMAIN DV DROP AMT DOSENO MDV CMT DROPPO DROPPA FORM II
ADDL PERIOD FORMGROUP PART ORDER


$DATA      data.csv IGNORE=#

$SUBROUTINE ADVAN13 TOL=6

$MODEL

; Oral

            COMP=(GUT) ;1. Gut

            COMP=(CENT) ;2. Central

            COMP=(PERI) ;3. Peripheral

; Patch

            COMP=(SKIN) ;4. Skin

            COMP=(DEPOT) ;5. Depot,from skin,transit


$PK


; Oral comp. params ==========

KA = EXP(LOG(THETA(1)) + ETA(1)) ; Absorption rate

CL = EXP(LOG(THETA(2)) + ETA(2)) ; Central clearance

VC = EXP(LOG(THETA(3)) + ETA(3)) ; Vd central

VP = EXP(LOG(THETA(4)))          ; Vd peripheral


Q = EXP(LOG(THETA(5)) + ETA(4)) ; Inter-compartmental clearance
```

```
KCP = Q/VC

KPC = Q/VP

KE = CL/VC


; Patch comp. params =========

KT = EXP(LOG(THETA(6)) + ETA(5)) ; n + 1 / MTT

KF = EXP(LOG(THETA(7)))


$DES

; Oral compartment diff. equations ==========

DADT(1) = -KA*A(1) ; Depot (gut)

DADT(2) = KA*A(1) + KPC*A(3) - KCP*A(2) - KE*A(2) + KT*A(5) ; Central_comp

DADT(3) = KCP*A(2) - KPC*A(3) ; Peripheral_comp


; Patch compartment diff. equations =========

DADT(4) = - KF*A(4) ; Skin (formulation)

DADT(5) = KF*A(4) - KT*A(5) ; Depot, transit


$ERROR

IPRED = 0

IF(A(2).GT.0) IPRED = A(2)/(VC/1000) ; IPRED (scaled)


W       =  SQRT((THETA(8)**2) + (THETA(9)**2)*(IPRED)**2)

IRES    =  IPRED - DV

IWRES   =  IRES/W

Y = IPRED + W*EPS(1)
```

```
$THETA
 (0,0.729133) ; 1.KA
 (0,9.37819) ; 2.CL
 (0,195.809) ; 3.V central (L)
 (0,525.574) ; 4.V peripheral (L)
 (0,49.8386) ; 5.Q
 (0,0.0244914) ; 6.KT
 (0,0.027617) ; 7.KF
 (0,0.292747) ; 8.Additive error
 (0,0.185568) ; 9.Proportional error
$OMEGA
 0.303063 ; 1.IIV KA
 0.0424066 ; 2.IIV CL
 0.284047 ; 3.IIV VC
 0.026579 ; 4.IIV Q
 0.207241 ; 5.IIV KT

$SIGMA  1  FIX
```

Code S5. NONMEM Code for case 2 (fractal model).

```
;; 1. Based on: transit_comp_v1.1

;; 2. Description: case2, fractal model

;; x1. Author: Woojin Jung

;; 3. Label: Oral and patch admin combined into two-compartment model

$PROBLEM    CASE2

$INPUT      ID TIME INIT REMAIN DV DROP AMT DOSENO MDV CMT DROPPO DROPPA FORM II
ADDL PERIOD FORMGROUP PART ORDER


$DATA      data.csv IGNORE=#

$SUBROUTINE ADVAN13 TOL=9

$MODEL

; Oral

            COMP=(GUT) ;1. Gut

            COMP=(CENT) ;2. Central

            COMP=(PERI) ;3. Peripheral

; Patch

            COMP=(SKIN) ;4. Skin

            COMP=(DEPOT) ;5. Depot,from skin,transit


$PK CALLFL=-2


IF (NEWIND < 2 ) THEN ; Recognizes any kind of first record

TD = 0 ;

ENDIF


IF (DOSENO.GT.0) THEN

 TD = TIME ; Time dosing

ENDIF
```

```
; Oral comp. params ==========
KA = EXP(LOG(THETA(1)) + ETA(1)) ; Absorption rate
CL = EXP(LOG(THETA(2)) + ETA(2)) ; Central clearance
VC = EXP(LOG(THETA(3)) + ETA(3)) ; Vd central
VP = EXP(LOG(THETA(4)))          ; Vd peripheral

Q = EXP(LOG(THETA(5)) + ETA(4)) ; Inter-compartmental clearance

KCP = Q/VC
KPC = Q/VP
KE = CL/VC

; Patch comp. params =========
TAU = 0.0000001
H = THETA(6) * EXP(ETA(6)) ; fractal exponent
KT = EXP(LOG(THETA(7)) + ETA(5)) ; n + 1 / MTT
KF = EXP(LOG(THETA(8)) - H*LOG(TIME - TD + TAU))

$DES
; Oral compartment diff. equations ==========
DADT(1) = -KA*A(1) ; Depot (gut)
DADT(2) = KA*A(1) + KPC*A(3) - KCP*A(2) - KE*A(2) + KT*A(5) ; Central_comp
DADT(3) = KCP*A(2) - KPC*A(3) ; Peripheral_comp

; Patch compartment diff. equations =========
DADT(4) = - KF*A(4) ; Skin (formulation)
DADT(5) = KF*A(4) - KT*A(5) ; Depot, transit
```

```
$ERROR
IPRED = 0
IF(A(2).GT.0) IPRED = A(2)/(VC/1000) ; IPRED (scaled)

W       =  SQRT((THETA(9)**2) + (THETA(10)**2)*(IPRED)**2)
IRES    =  IPRED - DV
IWRES   =  IRES/W
Y = IPRED + W*EPS(1)

$THETA
 (0,0.914658) ; 1.KA
 (0,8.97291) ; 2.CL
 (0,243.65) ; 3.V central (L)
 (0,413.263) ; 4.V peripheral (L)
 (0,56.6586) ; 5.Q
 (0,0.894192,1) ; 6.H
 (0,0.0186256) ; 7.KT
 (0,0.398403) ; 8.KF
 (0,0.678153) ; 9.Additive error
 (0,0.16358) ; 10.Proportional error

$OMEGA
 0.236701 ; 1.IIV KA
 0.043698 ; 2.IIV CL
 0.162774 ; 3.IIV VC
 0.00557957 ; 4.IIV Q
 0.105941 ; 5.IIV KT
```

```
 0.0269217 ; 6.IIV H


$SIGMA  1  FIX
```

Code S6. NONMEM Code for case 3 (base model).

```
;; 1. Based on: run042

;; 2. Description: case3, base model

;; x1. Author: Woojin Jung

;; 3. Label: intramuscular injection

$PROBLEM    CASE3

$INPUT      ID TIME DV DROP AMT MDV CMT ROUTE DOSENO


$DATA      data.csv IGNORE=#

$SUBROUTINE ADVAN9 TOL=9

$MODEL

; Transit comp.

            COMP=(D_FAST) ;1. muscle (depot,fast)

               COMP=(D_SLOW)  ;2.  muscle  (depot,slow),transit  by  stirling  ap-
prox.


; Physical comp.

            COMP=(CENT) ;3. central (V1)

            COMP=(PERI) ;4. peripheral (V2)

$PK CALLFL = -2


IF (NEWIND < 2) THEN ; Recongnizes any kind of first record

T1 = 0

DOSE1 = 0

ENDIF


; Physical prms =================================

V1 = EXP(LOG(THETA(1)) + ETA(1))  ; Vd (central)

V2 = EXP(LOG(THETA(2)))  ; Vd (peripheral)
```

```
CL = EXP(LOG(THETA(3)))  ; clearance (central)

Q1 = EXP(LOG(THETA(4)) + ETA(2)) ; inter-comp-clearance (central to periph)

; fractal prms ==================================

H = THETA(5) ; fractal exponent

KA1 = EXP(LOG(THETA(6)) - H*LOG(TIME) + ETA(3)) ; absorption rate (fast release,
from muscle)

KA2 = EXP(LOG(THETA(7))) ; absorption rate (slow release, from muscle)


; Transit prms ==================================

MTT = EXP(LOG(THETA(8))) ; mean transit time

N = EXP(LOG(THETA(9)) + ETA(4)) ; number of the compartments


; Dose partitioning ==============================

FRAC = EXP(LOG(THETA(10)) + ETA(5)) ; fraction, to fast absorption


F1 = FRAC ; dose partition - fast abs

F2 = 0


; Transit equation ==============================

LNFAC = LOG(2.5066) + (N+0.5)*LOG(N) - N


; rate constants

KT = (N+1)/MTT ; transit rate constant

KE = CL/V1 ; elimination  rate, central

KCP = Q1/V1

KPC = Q1/V2


IF (DOSENO == 1) THEN
```

```
T1 = TIME

DOSE1 = AMT

ENDIF


$DES

IF   (TIME>=T1.AND.DOSE1>0)   IPT1   =   EXP(LOG((1-FRAC)*DOSE1)   +   LOG(KT)   +
N*LOG(KT*(TIME-T1)) - KT*(TIME-T1) - LNFAC)


INPT = IPT1


; IM compartment diff. equations ==============

DADT(1) = - KA1*A(1) ; muscle (depot, fast)

DADT(2) = INPT - KA2*A(2) ; muscle (depot, slow), stirling approx.


; physiological compartment diff. equations ==============

DADT(3) = KA1*A(1) + KA2*A(2) + KPC*A(4) - KCP*A(3) - KE*A(3) ; central (V1)

DADT(4) = KCP*A(3) - KPC*A(4) ; peripheral (V2)


$ERROR

C_P = A(3)/V1

IPRED = C_P

W = SQRT((THETA(11)**2)*IPRED**2)


IRES     =  IPRED - DV

IWRES    =  IRES/W

Y = IPRED + W*EPS(1) ; DV=concentration [ug/L] AMT =ug


$THETA
```

```
 (0,0.233098) ; 1.V1,cent

 (0,1.98936) ; 2.V2,peri

 (0,0.144596) ; 3.CL

 (0,0.408052) ; 4.Q1

 (0,0,1) FIX ; 5.h

 (0,0.0843365) ; 6.KA1

 (0,0.00170504) ; 7.KA2

 (100,135.009,400) ; 8.MTT

 (0,86.848) ; 9.N

 (0,0.137736,1) ; 10.FRAC

 (0,0.320653) ; 11.Prop.err,centr

$OMEGA

 0.141981 ; 1.IIV.V1

 0.170659 ; 2.IIV.Q1

 0.0410159 ; 3.IIV.KA1

 0.00476087 ; 4.IIV.N

 0.178755 ; 5.IIV.FRAC

$SIGMA  1  FIX
```

Code S7. NONMEM Code for case 3 (fractal model).

```
;; 1. Based on: run042

;; 2. Description: case3, fractal model

;; x1. Author: Woojin Jung

;; 3. Label: intramuscular injection

$PROBLEM    CASE3

$INPUT      ID TIME DV DROP AMT MDV CMT ROUTE DOSENO


$DATA      data.csv IGNORE=#

$SUBROUTINE ADVAN9 TOL=6

$MODEL

; Transit comp.

            COMP=(D_FAST) ;1. muscle (depot,fast)

               COMP=(D_SLOW)  ;2.  muscle  (depot,slow),transit  by  stirling  ap-
prox.


; Physical comp.

            COMP=(CENT) ;3. central (V1)

            COMP=(PERI) ;4. peripheral (V2)

$PK CALLFL = -2


IF (NEWIND < 2) THEN ; Recongnizes any kind of first record

T1 = 0

DOSE1 = 0

ENDIF


; Physical prms =================================

V1 = EXP(LOG(THETA(1)) + ETA(1))  ; Vd (central)

V2 = EXP(LOG(THETA(2)))  ; Vd (peripheral)
```

```
CL = EXP(LOG(THETA(3)))  ; clearance (central)

Q1 = EXP(LOG(THETA(4)) + ETA(2)) ; inter-comp-clearance (central to periph)

; fractal prms ==================================

H = THETA(5) + ETA(6) ; fractal exponent

KA1 = EXP(LOG(THETA(6)) - H*LOG(TIME) + ETA(3)) ; absorption rate (fast release,
from muscle)

KA2 = EXP(LOG(THETA(7))) ; absorption rate (slow release, from muscle)


; Transit prms ==================================

MTT = EXP(LOG(THETA(8))) ; mean transit time

N = EXP(LOG(THETA(9)) + ETA(4)) ; number of the compartments


; Dose partitioning ==============================

FRAC = EXP(LOG(THETA(10)) + ETA(5)) ; fraction, to fast absorption


F1 = FRAC ; dose partition - fast abs

F2 = 0


; Transit equation ==============================

LNFAC = LOG(2.5066) + (N+0.5)*LOG(N) - N


; rate constants

KT = (N+1)/MTT ; transit rate constant

KE = CL/V1 ; elimination  rate, central

KCP = (Q1/V1)

KPC = Q1/V2


IF (DOSENO == 1) THEN
```

```
T1 = TIME

DOSE1 = AMT

ENDIF


$DES

IF   (TIME>=T1.AND.DOSE1>0)   IPT1   =   EXP(LOG((1-FRAC)*DOSE1)   +   LOG(KT)   +
N*LOG(KT*(TIME-T1)) - KT*(TIME-T1) - LNFAC)


INPT = IPT1


; IM compartment diff. equations ==============

DADT(1) = - KA1*A(1) ; muscle (depot, fast)

DADT(2) = INPT - KA2*A(2) ; muscle (depot, slow), stirling approx.


; physiological compartment diff. equations ==============

DADT(3) = KA1*A(1) + KA2*A(2) + KPC*A(4) - KCP*A(3) - KE*A(3) ; central (V1)

DADT(4) = KCP*A(3) - KPC*A(4) ; peripheral (V2)


$ERROR

C_P = A(3)/V1

IPRED = C_P

W = SQRT((THETA(11)**2)*IPRED**2)


IRES     =  IPRED - DV

IWRES    =  IRES/W

Y = IPRED + W*EPS(1) ; DV=concentration [ug/L] AMT =ug


$THETA
```

```
 (0,0.356114) ; 1.V1,cent
 (0,2.61518) ; 2.V2,peri
 (0,0.17515) ; 3.CL
 (0,0.659805) ; 4.Q1
 (0,0.026835,1) ; 5.h
 (0,0.103596) ; 6.KA1
 (0,0.00179072) ; 7.KA2
 (100,136.31,400) ; 8.MTT
 (0,83.7549) ; 9.N
 (0,0.172219,1) ; 10.FRAC
 (0,0.112738) ; 11.Add.err,centr
$OMEGA
 0.0682854 ; 1.IIV.V1
 0.201086 ; 2.IIV.Q1
 0.0364168 ; 3.IIV.KA1
 0.00649553 ; 4.IIV.N
 0.163404 ; 5.IIV.FRAC
 0.926984 ; 6.IIV.H
$SIGMA  1  FIX
```

Code S8. NONMEM Code for case 4 (base model).

```
;; 2. Description: case4, base

;; x1. Author: Ngo Thi Lien, Woojin Jung

$PROBLEM    CASE4

$DATA       data.csv IGNORE=@

$INPUT      ID TIME AMT CMT DV LNDV MDV EVID AGE BWKG HTCM BMI GR DOSE DVMG=DROP

$SUBROUTINE ADVAN6 TOL=6

$MODEL      NCOMP=4 COMP=(DEPOT,DEFDOSE) COMP=(ABS) COMP=(CENTRAL) COMP=(PERIPH)

$PK


      Kinj = THETA(1)* EXP(ETA(1))


      KSS1 = THETA(2)

      FcRn = THETA(3)

      Ka1  = THETA(4)* EXP(ETA(2))

      Ka2  = THETA(5)* EXP(ETA(3))

      Kdeg = THETA(6)* EXP(ETA(4))


      CL   = THETA(7)* EXP(ETA(7))

      V3   = THETA(8)

      Q    = THETA(9)

      V4   = THETA(10)


      Kint = THETA(11)

      Rtot = THETA(12)

      KSS2 = THETA(13)


      Kq   = THETA(14)* EXP(ETA(5))
```

```
      Alag1= THETA(17)* EXP(ETA(6))


      S2    = V3

      S3    = V4

      Kel   = CL/V3

      Kpt   =  Q/V3

      Ktp   =  Q/V4


$DES

; Absorption compartments

                 DAA    =    A(2)-FcRn-KSS1                                   ;
nmol

        Afree =   0.5*(DAA+SQRT(DAA**2+4*KSS1*A(2))) ; free, nmol, absorption
site

                 Ct   =    A(3)/V3                                            ;
nmol/L

           D  =  Ct-Rtot-KSS2                       ; nmol/L

           CP =  0.5*(D+SQRT(D**2+4*KSS2*Ct))       ; nmol/L


       DADT(1)  =  -Kinj*A(1)                                  ;  nmol,  injection
site

      DADT(2) =  Kinj*A(1) - (Kdeg + Ka1)*Afree - Ka2*FcRn*Afree/(KSS1+Afree) +
CP*Kq*V3 ; Total, nmol, absorption site


; Plasma (central) compartment

         DADT(3)  =   Ka1*Afree  +Ka2*(A(2)-Afree)  +Ktp*A(4)-(Kel+Kpt)*CP*V3  -
Kint*Rtot*CP*V3/(KSS2+CP) - CP*Kq*V3  ; nmol


; Tissue comparment
```

```
          DADT(4)    =    Kpt*CP*V3    -    Ktp*A(4)                                    ;
nmol


$ERROR
      Ctot  = A(3)/V3
      DD    = Ctot-Rtot-KSS2
      Cfree = 0.5*(DD+SQRT(DD**2+4*KSS2*Ctot))

      IPRED = Cfree
      W     = SQRT(THETA(15)**2+THETA(16)**2*IPRED**2)
      IRES  = DV-IPRED
      IWRES = IRES/W
      Y     = IPRED + W*EPS(1)


$THETA
 (0,1.22078) ; Kinj
 (0,245.883) ; KSS1  nmol
 (0,746.31) ; FcRn  nmol
 (0,0.0167784) ; Ka1   1/h
 (0,0.0341106) ; Ka2   1/h
 (0,0.0262355) ; Kdeg  1/h
 (0,0.210158) ; CL    L/h
 (0,11.2214) ; V3    L
 (0,0.028996) ; Q     L/h
 5.06 FIX ; V4    L
 0.206 FIX ; Kint  1/h
 (0,2.16248) ; Rtot  nmol/L
 (0,14.0691) ; Kss   nmol/L
```

```
 0.00952 FIX ; Kup   1/h Plasma Flow rate
 (0,0.184202) ; add
 (0,0.115236) ; pro
 (0,0.313979) ; Alag
$OMEGA
 0.957656 ; 1_Kinj
 0.528287 ; 2_Ka1
 0.109461 ; 3_Ka2
 0.0686738 ; 4_Kdeg
 0.837292 ; 5_Kq
 0.303671 ; 6_Alag
 0.0535786 ; 7_CL
$SIGMA  1  FIX
```

Code S9. NONMEM Code for case 4 (fractal model).

```
;; 2. Description: case4, fractal

;; x1. Author: Ngo Thi Lien, Woojin Jung

$PROBLEM    CASE4

$DATA       data.csv IGNORE=@

$INPUT      ID TIME AMT CMT DV LNDV MDV EVID AGE BWKG HTCM BMI GR DOSE DVMG=DROP

$SUBROUTINE ADVAN6 TOL=6

$MODEL      NCOMP=4 COMP=(DEPOT,DEFDOSE) COMP=(ABS) COMP=(CENTRAL) COMP=(PERIPH)

$PK


      Kinj = THETA(1)* EXP(ETA(1))


      KSS1 = THETA(2)

      FcRn = THETA(3)

      Ka1  = THETA(4)* EXP(ETA(2))

      H = THETA(18)

      KF = EXP(LOG(Ka1) -H*LOG(TIME))


      Ka2  = THETA(5)* EXP(ETA(3))

      Kdeg = THETA(6)* EXP(ETA(4))


      CL   = THETA(7)* EXP(ETA(7))

      V3   = THETA(8)

      Q    = THETA(9)

      V4   = THETA(10)


      Kint = THETA(11)

      Rtot = THETA(12)
```

```
      KSS2 = THETA(13)


      Kq   = THETA(14)* EXP(ETA(5))

      Alag1= THETA(17)* EXP(ETA(6))


      S2   = V3

      S3   = V4

      Kel  = CL/V3

      Kpt  =  Q/V3

      Ktp  =  Q/V4


$DES
; Absorption compartments
                 DAA   =    A(2)-FcRn-KSS1                                  ;
nmol
         Afree =   0.5*(DAA+SQRT(DAA**2+4*KSS1*A(2))) ; free, nmol, absorption
site
                 Ct   =    A(3)/V3                                          ;
nmol/L
           D  =  Ct-Rtot-KSS2                        ; nmol/L

           CP =  0.5*(D+SQRT(D**2+4*KSS2*Ct))        ; nmol/L


       DADT(1)  =  -Kinj*A(1)                                  ;  nmol, injection
site
      DADT(2) =  Kinj*A(1) - (Kdeg + KF)*Afree - Ka2*FcRn*Afree/(KSS1+Afree) +
CP*Kq*V3 ; Total, nmol, absorption site


; Plasma (central) compartment
        DADT(3)  =   KF*Afree  +  Ka2*(A(2)-Afree)  +Ktp*A(4)-(Kel+Kpt)*CP*V3  -
Kint*Rtot*CP*V3/(KSS2+CP) - CP*Kq*V3  ; nmol
```

```
; Tissue comparment
           DADT(4)   =    Kpt*CP*V3   -   Ktp*A(4)                            ;
nmol

$ERROR
      Ctot  = A(3)/V3
      DD    = Ctot-Rtot-KSS2
      Cfree = 0.5*(DD+SQRT(DD**2+4*KSS2*Ctot))

      IPRED = Cfree
      W     = SQRT(THETA(15)**2+THETA(16)**2*IPRED**2)
      IRES  = DV-IPRED
      IWRES = IRES/W
      Y     = IPRED + W*EPS(1)

$THETA
 (0,0.72295) ; Kinj
 (0,426.482) ; KSS1  nmol
 (0,685.457) ; FcRn  nmol
 (0,0.024555) ; Ka1   1/h
 (0,0.0472069) ; Ka2   1/h
 (0,0.022881) ; Kdeg  1/h
 (0,0.22751) ; CL    L/h
 (0,11.2913) ; V3    L
 (0,0.0304478) ; Q     L/h
 5.06 FIX ; V4    L
 0.206 FIX ; Kint  1/h
```

```
 (0,1.58768) ; Rtot  nmol/L
 (0,11.595) ; Kss   nmol/L
 0.00952 FIX ; Kup   1/h Plasma Flow rate
 (0,0.198217) ; add
 (0,0.107837) ; pro
 (0,0.286036) ; Alag
 (0,0.276798,1) ; H,Fractal exponent
$OMEGA
 0.721661 ; 1_Kinj
 0.642134 ; 2_Ka1
 0.124918 ; 3_Ka2
 0.0427415 ; 4_Kdeg
 1.32123 ; 5_Kq
 0.329387 ; 6_Alag
 0.053764 ; 7_CL
$SIGMA  1  FIX
```

Code S10. NONMEM Code for case 5 (base model).

```
;; 1. Based on: anakinra

;; 2. Description: case5, base (anakinra)

;; x1. Author: Ngo Thi Lien, Woojin Jung

$PROBLEM    CASE5

$DATA       data.csv IGNORE=@

$INPUT      ID TIME AMT DV CMT MDV EVID AGE BWKG HTCM BMI DVMG=DROP

$ABBREVIATED COMRES=2

$SUBROUTINE ADVAN6 TOL=9

$MODEL      NCOMP=3 COMP=(DEPOT,DEFDOSE) COMP=(CENTRAL) COMP=(AUC)

$PK

      CL    = THETA(1) * EXP(ETA(1))       ; Apparent clearance of free Anakinra
from central compartment

      VP    = THETA(2) * EXP(ETA(2))       ; Apparent volume of distribution of
free Anakinra in central compartment

       KaA    = THETA(3)  *  EXP(ETA(3))         ;  Absorption  rate  constant  of
Anakinra from injection site

      KelA   = CL/VP                           ; Elimination rate constant of free
Anakinra from central compartment

      KdegA  = THETA(4)                        ; Degradation rate constant of free
Anakinra in central compartment

      Rtot   = THETA(5)                        ; Total amount (unbound- and bound-to
Anakinra) of IL1R

      KSSA   = THETA(6)                        ; QSS constant for interactions of
IL1R and Anakinra



      S2    = VP

$DES

  ; QSS approximations for IL1R-Anakinra interaction

         Ct = A(2)/VP                          ; Total concentration of Anakinra in
central compartment (nmol/L)
```

```
        D  = Ct-Rtot-KSSA                ; (nmol/L)

        CP = 0.5*(D+SQRT(D**2+4*KSSA*Ct)); Concentration of free Anakinra de-
rived from total Anakinra concentration in

                                          ; central compartment (nmol/L)

  ; Injection site

    DADT(1) = -KaA*A(1)

  ; Central compartment-Total Anakinra amount  (nmol)

    DADT(2) =  KaA*A(1) -KelA*CP*VP - KdegA*Rtot*CP*VP/(KSSA+CP)

    DADT(3) =  CP


$ERROR

      Ctot  = A(2)/VP

      DD    = Ctot-Rtot-KSSA

      Cfree = 0.5*(DD+SQRT(DD**2+4*KSSA*Ctot))

      IPRED = Cfree

      W     = SQRT(THETA(7)**2+THETA(8)**2*IPRED**2)

      IRES  = DV-IPRED

      IWRES = IRES/W

      Y     = IPRED + W*EPS(1)


      AUC   = A(3)


$THETA

 (0,9.61029) ; CL     L/h

 (0,19.4421) ; VP     L

 (0,0.167366) ; KA     1/h

 0.206 FIX ; Kint   1/h

 (0,1.67529) ; Rtot   nmol/L
```

```
 (0,0.520918) ; Kss    nmol/L

 (0,0.0606311) ; Additive ERROR

 (0,0.114068) ; Proportiona ERROR

$OMEGA

 0.010519  ;          CL

 0.152892  ;          V2

 0.0627383  ;         KaA

$SIGMA  1  FIX
```

Code S11. NONMEM Code for case 5 (fractal model).

```
;; 1. Based on: anakinra

;; 2. Description: case5, fractal (anakinra)

;; x1. Author: Ngo Thi Lien, Woojin Jung

$PROBLEM    CASE5

$DATA       data.csv IGNORE=@

$INPUT      ID TIME AMT DV CMT MDV EVID AGE BWKG HTCM BMI DVMG=DROP

$ABBREVIATED COMRES=2

$SUBROUTINE ADVAN6 TOL=9

$MODEL      NCOMP=3 COMP=(DEPOT,DEFDOSE) COMP=(CENTRAL) COMP=(AUC)

$PK

      CL    = THETA(1) * EXP(ETA(1))       ; Apparent clearance of free Anakinra
from central compartment

      VP    = THETA(2) * EXP(ETA(2))       ; Apparent volume of distribution of
free Anakinra in central compartment

       KaA    = THETA(3)  *  EXP(ETA(3))         ;  Absorption  rate  constant  of
Anakinra from injection site

      H = THETA(4) * EXP(ETA(4))

      KF = EXP(LOG(KaA) - H*LOG(TIME))

      KelA   = CL/VP                           ; Elimination rate constant of free
Anakinra from central compartment

      KdegA = THETA(5)                         ; Degradation rate constant of free
Anakinra in central compartment

      Rtot   = THETA(6)                      ; Total amount (unbound- and bound-to
Anakinra) of IL1R

      KSSA   = THETA(7)                        ; QSS constant for interactions of
IL1R and Anakinra


      S2    = VP

$DES

  ; QSS approximations for IL1R-Anakinra interaction
```

```
         Ct = A(2)/VP                     ; Total concentration of Anakinra in
central compartment (nmol/L)

         D  = Ct-Rtot-KSSA                ; (nmol/L)

         CP = 0.5*(D+SQRT(D**2+4*KSSA*Ct)); Concentration of free Anakinra de-
rived from total Anakinra concentration in

                                          ; central compartment (nmol/L)

  ; Injection site

    DADT(1) = -KF*A(1)

  ; Central compartment-Total Anakinra amount  (nmol)

    DADT(2) =  KF*A(1) -KelA*CP*VP - KdegA*Rtot*CP*VP/(KSSA+CP)

    DADT(3) =  CP


$ERROR

      Ctot  = A(2)/VP

      DD    = Ctot-Rtot-KSSA

      Cfree = 0.5*(DD+SQRT(DD**2+4*KSSA*Ctot))

      IPRED = Cfree

      W     = SQRT(THETA(8)**2+THETA(9)**2*IPRED**2)

      IRES  = DV-IPRED

      IWRES = IRES/W

      Y     = IPRED + W*EPS(1)


      AUC   = A(3)


$THETA

 (0,9.02825) ; CL     L/h

 (0,53.6953) ; VP     L

 (0,0.469732) ; KA     1/h

 (0,0.139228) ; H,fractal exponent
```

```
 0.206 FIX ; Kint  1/h

 (0,1.67483) ; Rtot  nmol/L

 (0,1.391) ; Kss   nmol/L

 (0,0.0293577) ; Additive ERROR

 (0,0.112586) ; Proportiona ERROR

$OMEGA

 0.0122006  ;         CL

 0.0396066  ;         V2

 0.0705846  ;        KaA

 1.23662  ;          H

$SIGMA  1  FIX
```
